# Development of Proteomics-Based Fungicides: New Strategies for Environmentally Friendly Control of Fungal Plant Diseases

**DOI:** 10.3390/ijms12010795

**Published:** 2011-01-21

**Authors:** Francisco Javier Fernández Acero, María Carbú, Mohamed Rabie El-Akhal, Carlos Garrido, Victoria E. González-Rodríguez, Jesús M. Cantoral

**Affiliations:** Laboratory of Microbiology, Faculty of Marine and Environmental Sciences, University of Cádiz, Pol. Río San Pedro s/n, 11510 Puerto Real, Spain

**Keywords:** proteomics, 2-DE, aptamers, fungicides

## Abstract

Proteomics has become one of the most relevant high-throughput technologies. Several approaches have been used for studying, for example, tumor development, biomarker discovery, or microbiology. In this “post-genomic” era, the relevance of these studies has been highlighted as the phenotypes determined by the proteins and not by the genotypes encoding them that is responsible for the final phenotypes. One of the most interesting outcomes of these technologies is the design of new drugs, due to the discovery of new disease factors that may be candidates for new therapeutic targets. To our knowledge, no commercial fungicides have been developed from targeted molecular research, this review will shed some light on future prospects. We will summarize previous research efforts and discuss future innovations, focused on the fight against one of the main agents causing a devastating crops disease, fungal phytopathogens.

## 1. Introduction

Historically, agriculture is one of the main human activities. New improvements in crop cultures have paved the way for new settlements in the south of Spain, such as the strawberries cultivars in Huelva or grapevine plants in Jerez. Spain now produces 27% and 35% of European tomatoes and strawberries respectively. Most of these crops are developed in greenhouses or under strict environmentally friendly regulations. In both cases, the selected culture option may increase the risk of fungal diseases.

Fungal plant pathogen comprises an important group of microorganisms that cause significant economic losses in agriculture around the world. They are able to infect any tissue at any stage of plant growth [1,2]. For example, pathogens, such as *Fusarium* spp., *Phythophora* spp., are able to infect plant roots as well as other parts, while *Botrytis cinerea* is able to infect green tissues in over 200 plant cultivars, and is also responsible for considerable damage caused to fruit during storage and distribution.

Traditionally, when pathogens are detected in the field, or to prevent their appearance between crop seasons, disease control is carried out using proven chemical treatments. These treatments, however, have many drawbacks, due to their negative impact on the environment and public health [3]. For example, the emergence of fungicide-resistant strains, de-registration of fungicides, and public concerns regarding the health and environmental impacts of agrochemicals, may all act to limit their application in the future [4,5]. Moreover, the producers themselves have imposed quality regulations for good agriculture practices, such as GlobalGap, or Naturchoice. These regulations modulate the amount of fungicide used by including control rules with the product.

In spite of the incredible amount of biological information about fungal plant pathogens, there is no commercial fungicide developed from a molecular approach. During recent years, proteomics technology has increased its presence in molecular studies, offering to the research community the opportunity to unravel complex sets of proteins. Proteomics is a high-throughput technology that allows an in depth study of the sets of proteins synthesized in a specific sample (microorganism, cell, tissue, *etc*.) at any specific moment (constitutive *vs.* virulent stage). By protein profile comparison between samples, the proteins involved in specific biological processes may be revealed. The main aim of these assays is that the protein are responsible for the detected phenotype, and not the genes encoding them [6]. Several studies have revealed significant differences between the amount of transcripts and the level of protein expression in *Saccharomyces cerevisiae* [5]. Recent analysis of plant pathogen secretomes revealed several proteins *i.e*. arabinofuranosidase, that had previously been found in proteomic studies in *Botrytis cinerea* and *Sclerotiniae sclerotiorum*, but had disappeared from genomic approaches [6,7]. The role of these enzymes during the *B. cinerea* infection process is currently under investigation. Proteomics is very useful for mapping proteomes, isolation of subproteomes and studying protein-protein interaction [8]. Moreover, proteomic studies are crucial in order to understand protein post-translational modifications (PTMs). There are more than 200 PTMs described, allowing us to identify between 10 and 100 different biological functions from each gene. This compares with around 32,000 different genes estimated in the human genome, due to alternative splicing, sequence deletions, and PTMs occurring during protein biosynthesis. It is estimated that the total human proteome consists of over a million different protein species [9].

Proteomic studies have various applications from discovering proteins expressed during infection, to vaccine candidates. One of the most interesting applications of the proteomics is its use in discovering new protein targets for drug design [5,10]. It involves the identification and early validation of disease-associated targets. Despite the fact that most of the current drug targets are proteins, less than 500 human proteins are actually being used as drug targets. In recent years, pharmaceutical research and development spending has increased, but the number of drugs has not [9]. USD 500 million and 10 years is required for a pharmaceutical company to bring a new compound to the market. Most of these advances have been applied to solve medical problems. The use of proteomics to search new targets and develop fungicides is still mostly unknown. Fungal phytopathogens are one of the most serious problems in agriculture. They produce large losses to the farmers during plant development, storage and distribution. *B. cinerea* alone is responsible for 10% of the global fungicide market, representing more than € 500 million. Moreover, public awareness of the use of chemicals in the food chain, plus the fungicide associated environmental problems, are modifying the rules for fungicide selection and design. In this review, we will summarize future prospects with regard to fungicide design based on target selection and identification using proteomic approaches.

## 2. A Brief Fungal Biology Review

Fungal phytopathogens show a tremendous level of versatility. No part of the plant structure is exempt from fungal infections, for example, sweet fruits, leaves, and roots. Moreover, all plant developmental stages may suffer a fungal attack as *in planta* during fruit distribution. This capacity is supported by a complex fungal life and infection cycle.

**Figure 1:**
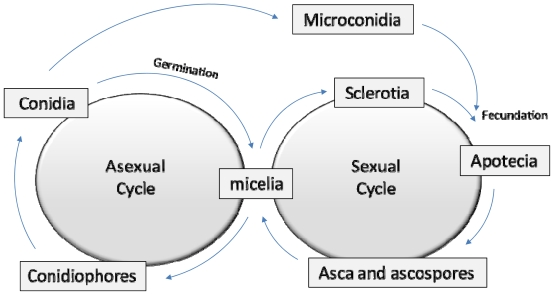
Schematic asexual and sexual cycles of ascomycete fungi.

Fungal plant pathogens have two different reproductive roles (Figure 1). During the asexual reproduction cycle (anamorphs) the fungus produces spores (conidia, zygospores, *etc.*). The sexual reproduction (teleomorphs) is mediated by the creation of sexual gametes, such as *B. cinerea* sclerotia and microconidia, which finally produce meiotic spores [11].

In most cases, fungal spores (asexual and meiotic) act as one of the main survival structures. These organisms are able to maintain their activity in soil or plant debris during winter, waiting for spring to germinate and infect a new harvest. Spores germinate through a “germ tube” after plant adhesion. To infect the plant, fungal species need to break through the plant cell walls. This step is mediated by the appressorium that produces a complex set of enzymes and toxins to disarm the plant defenses. When this is done, a thin invasion hypha penetrates into the plant tissues. To transform the plant biomass into fungal biomass, the thin hypha is transformed into a specific structure named haustorium, which is responsible for plant digestive degradation. Phytopathogenic fungi invade and colonize plant tissues, producing the first disease symptoms. The fungal mycelium then continues its invasion, regularly producing more asexual reproduction structures (*i.e*., conidiophores) to produce more spores that spread the disease as a secondary infection [12].

## 3. Pathogenicity and Virulence Factors

Fungal plant pathogens are a group of microorganisms that show a very high versatility during their infection cycles [1]. This versatility allows them to infect a wide variety of crops, and also the capacity to survive during unfavorable seasons, such as winter, staying ready to infect when the weather conditions become optimal [2]. They employ diverse strategies to infect and colonize the plants, and they also establish a complex interaction between fungus species and their hosts [13,14]. Common strategies phytopathogenic fungi use include forming specialized infection structures (*i.e.*, haustoria, appressoria, *etc*.) [13]; synthesis and secretion of different enzymes (*i.e.*, cuticule and cell wall degradation enzymes) [6,15]; production of secondary metabolites with antimicrobial properties (*i.e.*, phytoalexins, glycosides, glucosinolates, *etc*.) [13]; or even producing several families of toxins (*i.e.*, botrydial produced by *B. cinerea*, trichothecenes produced by *Gibberella* spp., *etc*.) [13,16–18].

Therefore, a considerable number of genes, which encode these proteins, metabolites and toxins, are involved in the infection cycles of phytopathogenic fungi. These genes have been named pathogenicity and/or virulence factors. To avoid misunderstandings of these terms, a pathogenicity factor is defined as a gene, protein or toxin that is necessary for the development of the disease, but is not essential for completing the pathogen life cycle *in vitro*. Pathogenicity refers to the capacity of a pathogen to cause disease. Virulence factors are those that are able to regulate the intensity of the infection, and virulence can be considered as the degree of pathogenicity of the corresponding gene, protein or toxin [1].

Over the years, advances in fungal molecular biology have allowed a better understanding of the strategies used by these phytopathogens. Traditionally, molecular genetic analysis identified an increasing number of pathogenicity/virulence factors in several fungal species [13,19–23], but more recently, modern development of proteomic techniques have accumulated a highly valuable quantity of information and several approaches leading to a broader are view of these fungal infection strategies [1,6,24–29].

The accumulation of information over the last decades, relating to i) fungal molecular genetic data, ii) pathogenicity/virulence factors and iii) proteomic approaches, has led to the appearance of several web-accessible databases which contribute to the fungal scientific community’s development in this field. More than 50 genomes of pathogenic fungi are published in the Broad Institute Database for public perusal (www.broadinstitute.org/science/projects/fungal-genomeinitiative); and further data in the Phytopathogenic Fungi and Oomycete EST Database, COGEME, (http://cogeme.ex.ac.uk/). Between these genera, there are human and plant pathogens, including important phytopathogenic species such as: *Aspergillus nidulans*, *A. tereus, Botrytis cinerea, Colletotrichum graminicola, Fusarium graminearum, F. oxysporum, F. verticillioides, Magnaporthe oryzae, M. grisea, Mycosphaerella fijiensis, M. graminicola, Nectria haematococca, Neurospora crassa, Puccinia graminis, P. triticina, Rhizopus oryzae, Sclerotinia sclerotiorum, Stagonospora nodorum, Ustilago maydis* and *Verticillium dahliae.* Pathogenicity and virulence factors are listed in The Pathogen-Host Interactions database (PHI-base, www.phi-base.org). This database contains more than one thousand entries, and therefore is a catalogue of experimentally verified pathogenicity, virulence and effector genes from fungal, Oomycete and bacterial pathogens, which infect animal, plant, fungal and insect hosts. PHI-base is an invaluable resource in the discovery of genes’ agronomically important pathogens. Most of the proteomic results achieved during the last years by several groups are also collected in several public databases. ExPASy (Expert Protein Analysis System) proteomics server of the Swiss Institute of Bioinformatics is a database dedicated to the analysis of protein sequences and structures as well as 2-DE approaches (http://www.expasy.org/). This database shows a proteomics-point of view of the fungal pathogens and adds to the PHI database, providing a whole understanding of the current information available on the pathogenicity/virulence factors of phytopathogenic fungi.

Knowledge of the pathogenicity/virulence factors essential for fungal infections is very important because it represents the targets that researchers must attack in the fight against these microorganisms. Proteomic techniques have contributed a high amount of data related to the proteins that fungi synthesize and secrete to the environment to complete their infection cycles [6]. Proteomic approaches, in which complex samples containing hundreds of proteins are identified in an experimental setup, offer a whole new perspective [10]. Therefore, Proteomics is a powerful potential tool for dissecting the molecular mechanisms underlying fungus-plant interactions [30]. Valuable knowledge is being obtained from these studies in the functional analysis of gene products and cellular pathways, and proteomics is being used to discover the proteins involved in particular disease stages [2].

*B. cinerea* is one example where advances have been achieved, especially due to its economical importance in crops losses. Many other advances have been achieved over the years, demonstrating the usefulness of proteomic techniques in identifying proteins of biological relevance in their infection cycles. In 2006, Fernández-Acero *et al*. [31] reported the first approach to the proteome analysis of *B. cinerea*. Up to four hundred protein spots were resolved in 2-DE after optimizing a protocol for protein extraction using phosphate buffer, followed by TCA-acetone precipitation. Due to the absence of genomic data on *B. cinerea*, the proteins had to be sequenced *de novo*, and 21 protein spots were positively identified. From this initial proteome map, most of the identified spots may play a crucial role as pathogenicity or virulence factors, including some housekeeping enzymes, such as malate and glyceraldehyde dehydrogenases [31]. In the last update of the proteome map of *B. cinerea* more than 300 proteins spots were identified by MALDI TOF/TOF, covering most of the known virulence factors [25,32] in this fungus.

As has been mentioned above, the determination of a specific factor as virulence or pathogenicity has been achieved by constructing defective mutants in the specific genes. In both cases, the infection power of the analyzed mutants should at least decrease or disappear compared to the wild types. If the deflections of these genes in mutants produce a loss of vegetal lesion, it is logical to assume that the inhibition of this enzyme or set of enzymes by targeted strategies, should produce new fungicides. In this context, the use of natural products or related compounds as specific enzyme inhibitors is an archetype, as they would be species specific and the environmental impact would be reduced to a minimum.

## 4. Classic Chemical Antifungal Biocides

“Antifungal biocides” is a general term to describe a chemical compound used to kill or inhibit fungi. Antifungal biocides are represented by a wide range of chemical agents. often well characterized in their behavior in diverse applications, also in clinical or agriculture fields, but frequently little understood in terms of the basis of their activity. It is often assumed that the same effects as those in non sporulating bacteria are responsible for fungal inactivation; the common view being that cellular effects occur by gross membrane damage, protein coagulation, or by cytoplasmic ‘poisoning’. In fact, it is believed that there is a common series of events starting with interaction at the cell surface followed by passage of a biocide through the fungal cell wall to reach its target site(s), but little information is available on the ways by which uptake into fungal cells is achieved, despite longstanding studies of biocide adsorption to yeasts and moulds [33]. There is thus a growing need to establish mechanisms of action for biocides to assist in the design of new compounds or combinations of compounds, in order to understand resistance mechanisms and to provide a focus for toxicological attention.

### 4.1. Mode of Action of Antifungal Biocides

Biocides comprise a heterogeneous group of chemical agents often well characterized in their behavior in diverse applications. Several biocides possess significant antifungal activities that depend on several factors, including concentration, pH, temperature, organic load, interfering substances, and the types of cells. The activity of biocides against fungal microorganisms is not as well documented as their activity against bacteria. In general, biocides are less active against filamentous fungi than against non-sporulating bacteria [34–37]. But, it is often assumed that fungal inactivation arises by mechanisms similar to, or identical with, those responsible for the destruction of non-sporulating bacteria [35,38]. Studies about the mode of action of antifungal biocides suggest that, unlike antibiotics for which selective action against specific cell targets is fundamental to their clinical value, biocides may act at one or several sites [39,40].

Antifungal substances target a range of cellular loci, from cell wall, plasma membrane to respiratory functions, enzymes and genetic material (Table 1). For example Aldehydes, such as gluteraldehyde, have broad spectrum of activity against fungi [40], which acts by virtue of its intermolecular cross-linking effects on amino groups in cell wall protein [41]. The presence of polymers, such as chitin on cell surfaces, indicates that this former is a potentially reactive site for cross-linking agents (gluteraldehyde and formaldehyde) [35]. Other reports indicated that gluteraldehyde agglutinates cells of *Candida lipolytica* and *Saccharomyces carlsbergensis* increase their settling rate on the outer cell layers as a result of using an antifungal agent [42].

For some antifungal agents, such as chlorhexidine, quaternary ammonium compounds (QACs), organic acids, esters and alcohols, cell membrane is probably their major target site. These biocides act by interfering with the structure or permeability of the cell membrane, subsequently altering its barrier function [35,38,43]. Thus, chlorhexidine induces leakage of intercellular materials and causes protoplast lysis. Chlorhexidine induces K^+^ loss from yeast and affects the ultrastructure of budding *Candida albicans* with loss of cytoplasmic constituents [44]. It has been observed that bisbiguandine inhibited filamentation probably as a result of some enzyme inhibition at the cytoplasmic membrane level [45]. Similarly, the toxic effect of some QACs agents against yeast cells resulted from disorganization of the plasma membrane, followed by inactivation of cell enzymes [46]. Ethanol disrupts the fungal plasma membrane of *S. cervisiae*, increasing the flux of protons across the membrane, disrupting the physiological function of the cell membrane [47], and inducing leakage of intracellular material [48]. Organic acids are rapidly taken up by yeast and act as membrane perturbers; their inhibitory effect is caused by the cell’s energetic commitment to restore a normal pH by altering its membrane properties and switching on an efflux pump system [49]. Esters are known as fungistatic agents and, at high concentrations, affect the plasma membrane causing leakage of intracellular constituents. However, at lower concentrations, they produce inhibition of the proton-motive force (ΔpH component) across the plasma membrane [50].

Strobilurin fungicide (QoIs), such as azoxystrobin and kresoxim-methyl, are the most important group of fungicide because they are highly effective against a wide variety of fungal pathogen. QoI target, cytochrome bc1, is an integral membrane protein complex, essential for fungal respiration. The fungicidal activity of QoIs relies on its ability to inhibit mitochondrial respiration by binding to the Qo site of the cytochrome bc1 complex, blocking electron transfer and halting ATP synthesis [51].

However, the DMI groups of fungicides, such as triadimenol, act by inhibiting demethylation at the 14-α carbon of lanosterol or 24-methylene dihydrolanosterol (eburicol), which are the substrates for the cytochrome P450-dependent 14-α demethylase in the biosynthesis of fungal sterols such as ergosterol [56]. For some agents, such as pyribencarb (benzylcarbamate-type fungicide), the effects of this fungicide is through inhibition of the electron transport system in fungi. It has been suggested that pyribencarb potentially inhibited succinate-cytocrome C reductase (SCR) activities of *B. cinerea, Corynespora cassiicola* and decylubiquinol-cytochrome C reductase (UCR) activity of *B. cinerea* in the same way as strobilurin fungicides [57].

Benzimidazole fungicides, such as benomyl, act through specific binding of the β-tubulin subunit of fungal tubulin, which consequently interferes with microtubule assembly, which in turn is essential for numerous cellular processes, such as mitosis and cytoskeleton formation [61]. The antifungal activity of heavy metals might be similar against bacteria [62]. Metal ions such as copper (Cu^2+^) and silver (Ag^2+^) have been proposed to interact strongly with thiol groups in fungal enzymes and proteins. The inhibitory activity of these compounds may be caused by protein (enzyme) damage through binding to key functional groups, particularly sulphydryl groups (-SH) in plasma membrane and within citosol [60].

Nucleic acid is probably a target site for some antifungal biocides such as flucytosine (pyrimidine analog). This agent is taken up by fungal cells via the enzyme cytosine permease. It is converted intracellularly first to 5-fluoroudidine (5-FU) and then to 5-fluorodeoxyuridine monophosphate (FdUMP) and fluorouridine triphosphate (FUTP), which inhibit DNA and RNA synthesis, respectively [59].

Further useful information on the mechanisms of antifungal action of biocides are reviewed in detail by Russell and Furr [59], McDonnell and Russell [59] and Russell [59]. Biocides exhibit a multiplicity of antifungal mechanisms. The knowledge of their mechanisms of action, combined with an understanding of quantitative structure-activity relationships, provides an important platform from which novel biocides may emerge, offering enhanced activity and environmental acceptability. Mechanisms of antifungal action of biocides may also be an important aid to our understanding, at a molecular level, the fungicidal mechanisms of resistance of a particular class of chemicals.

### 4.2. Mechanisms of Fungal Resistance to Biocides

**Figure 2:**
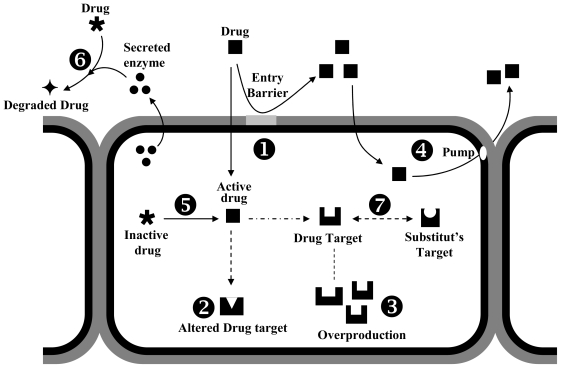
Mechanisms by which fungal cells might develop resistance (adapted from Ghannoum and Rice, 1999) [67]. **1**. The entry of the drug is prevented at the cell wall level. **2**. The drug target is altered so that the drug cannot bind to the target. **3**. The target enzyme is overproduced so that the drug does not inhibit the biochemical reaction completely. **4**. The drug is pumped out by an efflux pump. **5**. Some fungal enzymes that convert an inactive drug to its active form are inhibited. **6**. The cell secretes some enzymes to the extracellular medium which degrade the drug. **7**. The synthesis of an alternative enzyme, which replaces a drug target.

Resistance to biocidial agents has been widely studied in bacteria. However, very little is known concerning mechanisms of resistance to these chemical agents in fungi [35]. In order to survive biocide exposure, the main objective of the fungi is to decrease the toxic concentration of these chemical compounds, and for this aim, several resistance mechanisms are activated [63–66], including: (1) an altered target site, which reduces the binding of the fungicide; (2) the synthesis of an alternative enzyme capable of substituting the target enzyme; (3) the overproduction of the fungicide target; (4) an active efflux or reduced uptake of the fungicide; and (5) a metabolic breakdown of the fungicide (Figure 2). Some of these mechanisms are intrinsic to fungi, whereas others can be acquired. Acquired resistance can arise from mutation in one or several target sites genes. In addition, some unrecognized mechanisms could also be activated to confer cells with fungicide resistance [40].

The most common mechanism appears to be alteration of target site, particularly as a defence against single site of action fungicides. For example *Mycosphaerella fijiensis* is resistant to Qol fungicides; due to a single nucleotide change resulting in one amino acid (glycine) being replaced by another (alanine) in the target protein of the Qol fungicides, cytocrome B. This presumably disrupts the binding of fungicide to the protein, rendering the fungicide ineffective [51]. In *V. nashicola, B. cinerea* and *Gibberella fujikuroi*, the binding of 14C-carbendazim to tubulin-like proteins was much lower in benzimidazole-resistant isolates than in benzimidazole sensitive isolates, suggesting that a decreasing affinity of the fungicide to the target protein is a major factor in the resistance [61].

Upregulation of target genes can also render the biocide ineffective. This is seen in several DMIs resistant fungi. As it was shown in the previous section, the DMIs act inhibiting the sterol C-14 α-demethylation of 24-methylenedihydrolanosterol, a precursor of ergosterol in fungi [68]. It has been demonstrated that changes in the expression level of 14 α-demethylase (CYP51) gene might contribute to the gradual development of demethylation inhibitors (DMI) resistance [69]. In *C. glabrata*, the expression of CYP51 increased, which was responsible for DMI resistance. This fact resulted from an increase in number of copies of the CYP51 [70]. However, in some resistant isolates of *V. inaequalis*, high expression of CYP51 resulted from the presence of 553-pb insertion located in the promoter region [71]. It ha also been reported that the presence of a 126-bp repeat unit in the promoter region was responsible for CYP51A1 overexpression in DMI resistant *Penicillium digitatum* strains. Recently, the overexpression of ABC (ATP-Binding Cassette) transporters, which conferred DMI resistance, has been described in resistant isolates of *B. cinerea* and *M. graminicola* [72]. In clinical fungi, such as *C. albicans*, overexpression of ATP-dependent efflux pumps CDR1 and CDR2 confer cross-resistance to all azole antifungals [73].

Resistance to biocides can also be developed by efficient efflux of fungicide out of the cells. This system enables fungi to survive the exposure to toxic compounds by preventing their accumulation to toxic concentrations inside fungal cells. These membrane-bound proteins are known to provide protection against a wide range of toxic compounds [19]. The family of ATP-binding cassette (ABC) transporters and the major facilitator superfamily (MFS) are the most important efflux pumps involved in the protection of fungi against biocides [74]. *Septoria tritici* has developed multiple drug resistance using this mechanism; the pathogen had five ABC type transporters with overlapping substrate specificities, that work all together effectively pumping toxic chemicals out of the cells [72].

In *Aspergillus nidulans*, the energy dependent efflux of the DMI fenarimol was inhibited by compounds such as cycloheximide, cyclosporin and nigericin, suggesting that ABC transporters may affect the uptake/efflux balance of this fungicide. It has been shown that ABC transporters are involved in protection against compounds from all major classes of fungicides; including strobilurins [75,76].

In addition to the mechanisms outlined above, fungi may also develop metabolic pathways that circumvent the target protein, or acquire enzymes that enable metabolism of the fungicide to a harmless substance. For example, the presence of formaldehyde dehydrogenase in some fungi has been responsible for fungal resistance to formaldehyde [77]. In *C. albicans*, the resistance to flucytosine has been associated with alteration in cytosine deaminase, which results in a decrease in intracellular conversion of flucytosine to its active form. Recently, Hundt *et al*. [78] reported two moulds, *Trametes versicolor* and *Pycnoporus cinnabarinus*, which could metabolize a low concentration of bisphenol triclosan. Other fungi have been shown to degrade other diphenyl ether or biphenyl and phenols [78]. The resistance to heavy metals (including nickel, copper and mercury) has also been reported in fungal microorganisms [79]. In some strains of *S. cerevisiae*, the reduction of heavy metals toxicity has been attributed to hydrogen sulfide production which combines with heavy metals to form insoluble sulfides, rendering the yeast more resistant to copper and mercury [80]. Furthermore, in some nickel-resistant mutants of yeast and filamentous fungi, the resistance mechanisms involved inactivation of nickel toxicity by the production of extracellular nickel-chelating substances such as glutathione. A similar metal-resistance mechanism was found by Murphy *et al*. [81] with Cu^2+^-tolerant *A. niger, P. spinulosum, V. psalliotae* and *P. placenta*, which excreted a large amount of oxalate into a Cu-supplemented medium and detoxified the Cu by the formation of an extracellular Cu-oxalate complex.

Resistance to fungal biocide agents may also be acquired through one or several mutations. However, unlike bacteria, there is no evidence linking the presence of plasmids and other transferable genetic materials. Acquisition of biocides resistance has been observed in several [82] fungi species including *Candida* spp*., C. albicans, C. glabrata, C. dubliniensis, C. tropicalis*, *A. fumigatus* [83] and other agricultural fungi. In *B. cinerea*, specific resistance to strobilurins was correlated with a single mutation of the cytochrome B target gene in the respiratory system. In addition, specific resistance to carboxamides was also associated to mutations within the sdhB and sdhD genes encoding the iron-sulfur protein and an anchor protein of the succinate dehydrogenase complex [84].

The overall conclusion of the current evidence is that biocides resistance can be conferred by various mechanisms, but the most common resistance mechanism of phytopathogenic fungi to biocides is an alteration of the biochemical target site of the biocide. However, several other unrecognized mechanisms could also be activated to confer cells a fungicide resistance. Thus, research is greatly needed to increase our understanding of the molecular and biochemical mechanisms of resistance to chemical agents.

## 5. Basic Proteomics Workflow

Nowadays, proteomics platforms are supplying relevant biological information to the research community. These efforts are named the actual molecular biology as “the post-genomic era”. The term proteomics [85] includes a set of techniques to study the complete set of proteins expressed by a specific microorganism, cell, tissue, *etc.* in a specific sample moment. The first characteristic of proteome analysis is that it is highly dynamic, obtaining infinite proteomes from a specific genome. Despite several techniques to solve this problem, we would like to highlight that the experimental design plays a crucial role in these assays. The obtained proteome output is clearly related to the input conditions. The obtained conclusions of our experiment about biological relevance are determined by experimental set up; this is the first proteomic procedure.

**Figure 3:**
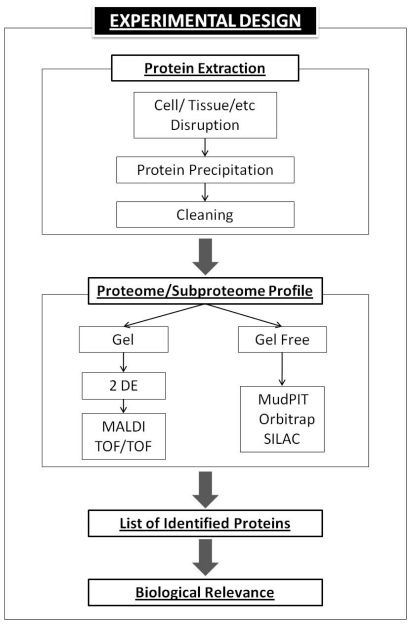
Schematic view of a typical proteomic experiment.

For the novice proteomics user, here is a brief route of a basic proteomic experiment (Figure 3). Due to proteomics work with proteins, we must optimize protocols to obtain protein extracts with a high degree of quality. Impurities must be extracted, such as salts, DNA, proteases *etc*. which are able to seriously disturb the experiment and avoid, for example, protein movements during isoelectrofucusing (IEF). Depending on the biological sample used, protein extraction protocols have three different steps. The initial step is the cell or tissue disruption (i) to break the cell walls and membranes, *i.e.*, pestle and mortar in the presence of liquid nitrogen has been widely used. Then, the proteins are precipitated (ii) normally with trichloroacetic acid (TCA) plus acetone. And finally, protein pellets are cleaned (iii) to remove impurities *i.e.*, with acetone, water or phenol.

After protein extraction, we must apply a specific separation procedure to be able to study and/or compare the proteomes. Currently, two main technologies are used, gel or gel-free proteomic platforms. The most common technique for separation and the study of the components of the proteoma is the two-dimensional poly-acrylamide gel electrophoresis (2-DE). Proteomics studies based on 2-DE gel electrophoresis has been widely used. Protein extracts are separated by using two different criteria; first by its isoelectric point (pI) and by its molecular weight (mW). This allows the separation of large-scale proteins, and has great resolution power in compound mixtures of proteins.

After protein separation, gels are stained. This step is crucial since it determines the type of study that we want to accomplish. These staining systems may be specific (antibodies or those associated to posttranslational modification, *i.e.*, pro-Q Diamond) or unspecified (Comassie, Brillient blue, Sypro, *etc.*). Fundamentally, two main staining criteria must be taken into account: the level of sensibility and its compatibility with mass spectrometry. Gels are digitalized and spots are studied by specific software (*i.e.*, PD Quest, Bio-Rad). Spots of interest are scised from the gels and prepared for mass spectrometry studies. As an example, protein spots are digested with trypsin and the peptide mixture studied by MALDI TOF (Matrix Assited Laser Desorption Ionization-Time of Flight) to obtain the peptide masses or PMF and/or MALDI TOF/TOF to obtain the peptide fragmentation fingerprinting (PFF) and obtain peptide sequences. There are several instruments to analyze protein peptides. Proteomics has been developed in parallel to the improvements of the mass analyzer. Recently, gel-free proteomic systems have been evolved. In brief, in these systems, proteins are digested directly after extraction. Peptides are separated by using coupled chromatography columns (2-D chromatography). Normally it uses cation exchange plus reversed phase columns (MudPIT, Multidimensional Protein Identification Technology). It allows that peptides can be interfaced directly with the ion source of a mass spectrometer. These assays are also called “shotgun proteomics” due to the fact that they are able to obtain a lot of protein identifications without using the labor intensive 2-DE method. Based on this technology, specific protocols to tackle quantitative experiments have been developed (SILAC). New improvements in mass analyzers, such as the Orbitrap instrument enhance technologies to a new advanced level or second generation of proteomics.

Protein identification by MS or MS/MS analysis results in a list of identified proteins, with name, accession number, percentage of protein coverage, ID scores, *etc*. Some informatics resources have been implemented; gene onthology has been used to classify protein identification into its biological function or its involved biological processes. Most of the proteomic analyses identified a long list of proteins; the biological relevance of this identification in any specific process must be retained for further specific approaches. However, significant information for drug discovery has been highlighted [1,2].

## 6. Bioinformatic Approaches

As has been previously mentioned, the result of a proteomic experiment is a long list of identified proteins, related or not to a specific biological question. Mostly further analysis is then necessary to check the role of specific proteins by gene silencing or knock-out mutant generation. However, several bioinformatic resources have been created to compile relevant information solely by using the peptide sequence. We are therby able to predict the protein secondary structures, 3D characterization and protein localization in the cell, or its interaction with other proteins or compounds. Apart from the usefulness of relevant protein information from existing databases, the potential use of these sequences to check the suggested role of a specific protein as a therapeutic target remains undilucidated.

Proteomic techniques are able to separate and characterize complex sets of proteins. Moreover, the vast majority of current drug targets are proteins. As well as using proteomics to settle drug targets of a specific compound or to search new therapeutics objectives [1], the development of mathematical algorisms to predict its role may be a useful tool to drug discovery through proteomics analysis. Xu *et al.* (2007) [86] presented the concept of “drug target-likeness” of a protein as an independent set of characteristics of successful targets. By a thorough study of known drug targets, it is possible to determine if an obtained protein sequence fits with this drug target role [87].

This methodology may open a new frontier in fungicide design. However, the papers described tried to find shared features of human disease targets, by the assumption of that (i) structurally similar (oral drug-like) chemicals might require similar target structures; and (ii) good therapeutic index might be achieved by regulating proteins that are specific to diseases (not causing severe toxicity), robust (less affected by individual genomic compositions), and effective (not in a complex homeostasis network). Therefore, good human targets may display shared characteristics in sequence (structure), function, network connectivity*,* single nucleotide polymorphisms (SNP), *etc*. However, these concepts are not seemingly generalizable to plant fungicide design (Xin Chen, *pers. communication*). In so far as this algorithm applys to fungal plant pathogens, the accumulation of molecular information on these organisms, and the commercial interest of developing environmentally friendly fungicides, will press the research community to improve mathematical algorisms to predict the role of a protein as a fungicide.

## 7. New Protein-Based Strategies to Classical Chemical Fungicide Design

Historically, drugs have been obtained from plant and animals products, from derivates of human endogenous ligands or from chemicals or semi-synthetic chemicals. Classical methods to control fungal plant diseases are based on the use of chemical compounds. In spite of the success achieved, new criteria for the indiscriminate use of toxic compounds in nature avoid using this technology. Control strategies based on classical fungicides produce serious collateral effects, mainly related with environmental pollution and the development of multidrug resistance.

Several changes in the design of chemical fungicides are being tackled by the research community by summarizing the genomic and proteomic information available. Biosynthetic fungicide design has been established as a new focus in fungicide development [88]. Based on an in depth study of fungal biology, the use of alien or modified natural compounds provides a potential species-specific method of controlling plant pathogens by specific inhibition of those proteins involved in the infection cycle [89]. The use of these compounds minimizes the environmental impact as they are biodegradable, possess high specificity, and poorly integrate in the food chain.

In the post-genomic era, new terms related with chemical “-omics” have appeared. The term “genetic chemical” describes the use of small molecules to selectively perturb gene function. When this concept is applied on a genome-wide scale it is named “chemogenomics”. The application of chemogenomics to protein targets is named “chemoproteomics”; although a more explicit definition is TRAP (targeted related affinity profiling) defined as the use of biology to inform chemistry [90]. The accumulation of proteomic information of fungal plant pathogens may be an incentive to the development of new and environmentally friendly fungicides.

## 8. Peptide Aptamers

Since chemical strategies produce several problems, new molecular approaches focused on targeted design are being developed. One of the most promising technologies is the use of peptide sequences that are able to modify protein activities. Those “aptamers” will be able to disrupt fungal development, or strengthen plant immunity, by interacting with specific proteins obtained from proteome mining approaches. Peptide aptamers are molecules from 8 to 20 amino acids defined by their ability to bind to specific proteins, potentially inhibiting or activating them.

These peptides are inserted as a part of the primary sequence of a structurally stable protein, called a scaffold. Functional peptide aptamers have properties similar to antibodies [91], presenting similar dissociation constants. Moreover, they can be used as antibodies in several applications such as nitrocellulose immunoblots. Peptide aptamer libraries have until now been obtained from (i) two yeast hybrid libraries, (ii) yeast expression libraries, (iii) bacterial expression libraries, and (iv) retroviral libraries for expression in mammalian cells [92]. Normally, selection is randomized by following the binding capacity to specific molecules. However, it can be designed to bind specific substrates. Nowadays, the validation of candidate drug targets that are being identified by proteomic approaches can clearly be improved by this technology which can determine the role of each candidate in a given pathology.

Peptide aptamers have primarily been used as research tools to manipulate protein function and study regulatory networks. However, its potential use in therapeutic research has been validated for target identification and validation to drug discovery. To apply this powerful technology, we only need a set of proteins involved in fungal development or plant defense, and proteomics will be the primary source of this information. Even though the use of these technologies in crop fields is difficult, some previous results [91] in this agronomic area are optimistic. Using this approach, a novel strategy to develop virus-resistant plant to tospovirus has been presented by the transgenic expression of an interfering peptide. The use of these compounds to inhibit the spreading of fungal plant diseases may soon be a viable option, since these diseases are most prevalent in world crops.

## 9. RNA Silencing

Quelling is a gene silencing phenomenon that belongs to the RNA-mediated gene silencing mechanism, which has been defined as the reversible inactivation of gene expression. This technique has been widely used in molecular biology studies to produce the “knockdown” (silence) of specific genes and explores its role in several biological processes.

Two major RNA silencing pathways have been identified in animals and plants, namely, the small interfering RNA (siRNA)-directed pathway and the microRNA (miRNA)-directed pathway. This machinery has not been found in the prokaryotic kingdom *Monera*, and may have emerged before the divergence of the major eukaryotic lineages. This process has been studied in several phytopathogenic fungi [93], but its use in controlling plant disease is still unexplored.

RNA silencing opens new routes to explore fungal biology. In comparison with knock-out strategies, knock-down strategies present several advantages. RNA silencing solves the problem of low efficiency of homologous recombination found in the knock-out strategies in several microorganisms. At the same time, this strategy also solves the problem found in multinucleate cells of fungi. Moreover, it allows gene inactivation in a specific stage, tissue, *etc*. because the gene is not eliminated permanently. Alternative mRNA spliced isoforms or lethal genes may be studied by knock-down protocols [94]. Disadvantages of this process are related to the residual protein production in knock-down strains, suggesting that the silencing is not absolute.

The use of RNA silencing technologies as a tool in the development of new drugs, with a high level of specificity against specific protein targets developed on the base of proteome mining profiling, is a promising technology. Some patents of ocular diseases treatment have been approved [95]. The use of this technology to unravel fungal pathology processes is increasing. However, their potential uses in crop treatments are in the initial stages.

## 10. Peptide Probes

As has previously been mentioned, the huge amount of information obtained from proteomics studies needs to be thoroughly analyzed to unravel the significant biological relevance hidden behind protein identification. One of the most promising strategies in the fight against fungal diseases, is the use of a novel chemical proteomics tool, called activity based protein profiling (ABPP) [96]. This technology allows us to reveal activities of proteomes and it will therefore be crucial to uncover an essential layer of information in biological processes by displaying the activities of protein classes (www.plantchemetics.org). ABPP uses small-molecule fluorescent probe that irreversibly reacts with the catalytic site of the catalytic subunits in an activity-dependent manner. Activities can be quantified from fluorescent protein gels and used to study activities *in vitro* and *in vivo* [97].

The potential use of this technology to suppress protein activities is being developed, named “targeted chemical genetics”. It could be useful to unravel the role of specific enzymes in biological or pathogenic processes. However, the inhibition of certain specific gene products or its PTMs by knock-out mutants resulted in a loss of fungal pathogenicity. This happened because the selected protein was a pathogenicity factor. The potential use of this technology to monitor fungal diseases, or to develop new environmentally friendly fungicides seems clear.

## 11. Conclusions

In the present post genomic era, proteomics has displayed a crucial and potent role in tackling the existing “black hole” between genotypes and phenotypes. The number of published researches and existing technologies are increasing continuously. Significant advances have been achieved in several fields, such as medicine or microbiology. One of the most interesting applications of this technique is the development of new strategies for drug design.

At present, a vast amount of the used drug targets are proteins. These advances allow the development of new drugs with a high level of specificity, oriented to inhibit or modify specific protein isoforms or/and enzyme activities. In spite of these advances having significant and interesting results for human medicine approaches, there has been no application of these technologies in fungicide design. Plants pathogenic fungi are one of the most devastating plant diseases producing relevant losses to farmers during plant growth, fruit distribution and storage. Moreover, the indiscriminate use of chemicals has a profound impact on the environment. It is necessary to develop new strategies to attack plant pathogen without a detrimental impact on nature. Proteomics can identify the pathogenicity/virulence factors. In this review we have described existing techniques to develop fungicides based on proteomic data. In spite of significant results these technologies have produced in several molecular biology areas, their use against fungal infection constitutes, in some cases, a science fiction exercise. Other technologies described have been used with varying degrees of success, such as the design of new chemical fungicides. However, the wide use of proteomics in crop fields remains unclear. Such approaches could be used in combination with other environmentally friendly initiatives to control fungal plant diseases in order to minimize the likeliness of resistance in fungal species. The time when these techniques will be common for farmers is near, due to the fact that environmental protection rules already prohibit the free use of fungicides in the E.U.

## Figures and Tables

**Table 1:** Target sites and mechanisms of antifungal action of some biocides.

Target site	Mechanisms of action	Example biocides	References
Cell wall	1. Cross-linking of cell proteins and chitin	Gluteraldehyde	[35,41,52]
	2. Cell agglutination		[42]
Plasma membrane	1. Induction leakage of intercellular materials and protoplast lysis; loss of structural organization and integrity; disruption to physiological function	Chlorhexidine, QAC’s Ethanol	[53] [46] [47] [48]
	2. Alteration of membrane properties and switching on an efflux pump system; membrane perturbation	Organic acids	[54] [55] [49]
	3. Inhibition of the proton-motive force (Δcomponent); induction leakage of intercellular materials	Esters	[50]
	4. Inhibition of respiration and energy transfer; inhibition of ATP synthesis	QoI’s	[51]
	5. Interaction with ergosterol and destabilization of cell membrane functions; inhibition of cytochrome P450 in ergosterol biosynthetic pathway	DMI groups	[56]
	6. Inhibition of the electron transport system	Benzylcarbamate	[57]
DNA/RNA	Interferes with DNA and RNA synthesis	Pyrimidine analog: Flucytosine	[58,59]
Protein	1. Interaction with alkylating and oxidizing agents; binding to key functional groups of fungal enzymes	Heavy metals (-SH groups)	[60]
	2. Inhibition of cell division; bind to proteins of tubulin; cytoskeleton formation	Benzimidazole	[61]
